# Cardiac injury modulates critical components of prostaglandin E_2_ signaling during zebrafish heart regeneration

**DOI:** 10.1038/s41598-020-59868-6

**Published:** 2020-02-20

**Authors:** MaryLynn FitzSimons, Megan Beauchemin, Ashley M. Smith, Erika G. Stroh, Daniel J. Kelpsch, Maureen C. Lamb, Tina L. Tootle, Viravuth P. Yin

**Affiliations:** 1 Kathryn W. Davis Center for Regenerative Biology and Medicine, MDI Biological Laboratory, Bar Harbor, ME 04609 US; 2 Graduate School of Biomedical Sciences and Engineering, University of Maine, Orono, ME 04469 US; 3 Anatomy and Cell Biology, Carver College of Medicine, University of Iowa, Iowa City, Iowa 52242 US; 4 Present Address: The University of New England, Biddeford, ME 04005 US; 5 Present Address: Department of Embryology, Carnegie Institution for Science, Baltimore, MD 21218 US

**Keywords:** Stem-cell niche, Regeneration

## Abstract

The inability to effectively stimulate cardiomyocyte proliferation remains a principle barrier to regeneration in the adult human heart. A tightly regulated, acute inflammatory response mediated by a range of cell types is required to initiate regenerative processes. Prostaglandin E_2_ (PGE_2_), a potent lipid signaling molecule induced by inflammation, has been shown to promote regeneration and cell proliferation; however, the dynamics of PGE_2_ signaling in the context of heart regeneration remain underexplored. Here, we employ the regeneration-competent zebrafish to characterize components of the PGE_2_ signaling circuit following cardiac injury. In the regenerating adult heart, we documented an increase in PGE_2_ levels, concurrent with upregulation of *cox2a* and *ptges*, two genes critical for PGE_2_ synthesis. Furthermore, we identified the epicardium as the most prominent site for *cox2a* expression, thereby suggesting a role for this tissue as an inflammatory mediator. Injury also drove the opposing expression of PGE_2_ receptors, upregulating pro-restorative *ptger2a* and downregulating the opposing receptor *ptger3*. Importantly, treatment with pharmacological inhibitors of Cox2 activity suppressed both production of PGE_2_, and the proliferation of cardiomyocytes. These results suggest that injury-induced PGE_2_ signaling is key to stimulating cardiomyocyte proliferation during regeneration.

## Introduction

Ischemic heart disease is currently the leading cause of mortality worldwide^1^. A fundamental limitation to cardiac regeneration in adult humans is the inability to effectively replenish lost cardiomyocytes (CMs). In stark contrast, multiple vertebrates, including the axolotl, newt, zebrafish, and neonatal mouse, demonstrate the remarkable capacity to regenerate lost or damaged myocardium through the proliferation of resident cardiac muscle cells^2–5^. Therefore, elucidating the cues that promote CM proliferation in these models will be crucial to informing therapies to stimulate regeneration in the injured human heart.

Acute inflammation is a hallmark of the tissue damage response, requiring the meticulous orchestration of signals from multiple cell types. CMs, endothelial cells, immune cells, and more recently, epicardial cells, have been shown to play essential roles in crafting the injury micro-environment^6–11^. A highly regulated inflammatory response is required for regeneration, and abrogating this response blocked regeneration in both zebrafish and neonatal mouse hearts^12–14^.

Among the principal signaling molecules released during acute inflammation are the prostaglandins. Notably, Prostaglandin E_2_ (PGE_2_) has been shown to promote tissue regeneration by directly stimulating the proliferation of target cells^15,16^, as well as indirectly, by governing the recruitment and polarization of immune cells^17–19^. PGE_2_ is synthesized through the sequential catalytic activity of cyclooxygenase (COX) enzymes, prostaglandin-endoperoxide synthase −1 and −2 (colloquially termed COX1 and −2), followed by the terminal synthase Prostaglandin E synthases (PTGES). There are four membrane-bound PGE_2_ receptors. Signaling through these receptors has pleiotropic effects; however upregulation of Prostaglandin E_2_ receptor 2 (EP2) and EP4 is frequently associated with cancer progression, cell survival and proliferation^20–24^, while activation of EP3 often stimulates opposing cellular responses^25–27^.

The multi-faceted effects of PGE_2_ activity are also mediated by the spatio-temporal dynamics of signal intensity. Although PGE_2_ has been demonstrated to accelerate skeletal muscle regeneration^28^, it also exacerbated ischemic damage in the brain, and inhibited repair and regeneration of the liver^29–31^. Of concern, PGE_2_-pathway genes are also upregulated in many cancers^32–35^, and while short-term PGE_2_ treatment promoted regeneration of damaged colon tissues, prolonged exposure-initiated tumor formation^36^. Therefore, it is critical to decode the precise dynamics of pro-regenerative PGE_2_ signaling to capitalize upon the benefits of this inflammatory mediator.

In the present study, we show that the regenerating zebrafish heart upregulates enzymes essential for PGE_2_ synthesis, and modulates PGE_2_ receptor expression to optimize pro-regenerative signaling. Moreover, we identify the epicardium as a focus of *cox2a* expression, implicating this tissue as a source of inflammation-associated PGE_2_ signaling. Importantly, pharmacological inhibition of Cox2 activity suppressed both levels of PGE_2_, and CM proliferation early in the regenerative process. Taken together, our data suggest that PGE_2_ promotes CM proliferation during the acute inflammatory response, and holds potential therapeutic promise for humans.

## Materials and Methods

### Zebrafish husbandry and heart amputation

All animal studies were performed in accordance with an approved protocol (Protocol 16–14) by the Institutional Animal Care and Use Committee at MDI Biological Laboratory. Wild type animals used were of the Ekkwill (EK) or EKxAB mixed background strains. Transgenic lines used in this study were *Tg(cmlc2:EGFP*)^37^, *Tg(tcf21:DsRed*)^38^, *Tg(fli1a:EGFP)*^39^, and *Tg(mpeg1:YFP*)^40^. Adult zebrafish 4–18 months of age were anaesthetized in a 1:1,000 dilution of 2-phenoxyethanol. Approximately 20% of the ventricular apex was resected with iridectomy scissors, as previously described^5^.

### Intraperitoneal microinjection of selective Cox2 inhibitors

Zebrafish were injected daily, beginning on the day of surgery, with 220 ng/g of NS-398 or 1 μg/g Celecoxib (Cayman Chemical, Ann Arbor, MI #70590, #10008672); vehicle controls were 0.1% DMSO, or 0.44% EtOH, respectively.

### Immunohistochemistry

Hearts were extracted, fixed in 4% paraformaldehyde, equilibrated in 30% sucrose, then embedded in TBS tissue freezing medium (Fisher Scientific, Hampton, NH #TFM-C) and sectioned to 10 μm. Primary antibodies used were rabbit anti-Mef2 (Santa Cruz Biotechnology, Santa Cruz, CA #SC-313; 1:75) and mouse anti-Pcna (Sigma-Aldrich, Nadick, MA #P8825; 1:400). Secondary antibodies used were Alexa Fluor 488 goat anti-rabbit IgG (H + L) for anti-Mef2, and Alexa Fluor 594 goat anti-mouse IgG (H + L) for anti-Pcna (Invitrogen - Thermo Fisher Scientific, Waltham, MA #A11034, #A11020). Images were captured at 20× using an Olympus BX53 microscope and Retiga 2000DC camera. CM proliferation indices were calculated as a percentage of Mef2(+)Pcna(+) cells relative to the total number of Mef2(+) cells in a defined area adjacent to the injury.

### *In situ* hybridizations

Hearts were processed as described above, and serial sections were subjected to *in situ* hybridization studies with DIG-labelled RNA probes directed against *cox2a*, *cox2b* and *ptger2a* using previously described methods^5,41^. cDNA fragments corresponding to the first 900-bp of each gene were synthesized (www.IDTDNA.com) and cloned into the pMiniT vector (www.NEB.com). Antisense probes were synthesized with either SP6 or T7 Polymerase using the Roche DIG Labelling Kit (SP6/T7) in accordance to the manufacturer’s suggested protocol (www.Roche.com). Negative controls included *cox2a* riboprobe but no secondary antibody, and secondary antibody only. Images were captured using settings described under the Immunohistochemistry methods section.

### Gene expression studies of whole heart tissues

Ventricles were isolated in ice-cold PBS, placed immediately in Ambion TRIzol Reagent (Invitrogen - Thermo Fisher Scientific, Waltham, MA #2302700), and homogenized using an electric homogenizer. RNA was extracted using Zymo Direct-zol RNA Microprep Kit, (Zymo Research Corp, Irvine, CA #R2069), followed by treatment with DNAseI (New England Biolabs, Ipswich, MA #M0303), and final purification with RNA Clean & Concentrator-5 Kit (Zymo Research Corp, Irvine, CA #R1014). cDNA was synthesized using ProtoScript^®^ II First Strand cDNA Kit (New England Biolabs, Ipswich, MA # E6560S). qPCR studies were performed in technical triplicate using Brilliant III Ultra-Fast SYBR^®^ Green QPCR Master Mix (Agilent, Santa Clara, CA #600882) and transcript specific primers (Table 1) in a Roche LightCycler^®^ 480. Relative gene expression was determined using the 2^−ΔΔCt^ method and normalized to the reference gene *rpl13a*. Fold-change was expressed on a log_2_ scale.

**Table 1:** Gene expression primer sequences.

Gene	Sequence (5′ to 3′)
cox1 FWD	CGGAAAGTGCTCACAGTAAGA
cox1 REV	CTGGTGTAGTAGGTGATGTTGG
cox2a FWD	AACTCTATCGTCACCAC
cox2a REV	CCTGTCATCTCCTCAAA
cox2b FWD	GGCTCATCCTTATTGGTGAGACTAT
cox2b REV	TCGGGATCAAACTTGAGCTTAAAATA
ptger2a FWD	TGCGGATACATCACCATCCCTTGT
ptger2a REV	GTGGCGTAAACATTGGCATACGCT
ptger3 FWD	TGATGGTCACTGGAATGGTGGGAA
ptger3 REV	TCCACGCGGTCCCATTTCATATCT
ptger4a FWD	TGCCAATATTTCGGCTTCGTGCTG
ptger4a REV	ATGCGTAAATGGCGAGTAGGGTGA
ptges FWD	CATATGTGGAGCGCTGTAGG
ptges REV	GATGGGCTTGTCATGGAGTAG
rpl13a FWD	TCTGGAGGACTGTAAGAGGTATGC
rpl13a REV	AGACGCACAATCTTGAGAGCAG

### Fluorescence-Activated Cell Sorting (FACS) and gene expression

Single-cell suspensions were prepared from 12–45 hearts of zebrafish reporter strains as previously described, with modifications^42^. Briefly, ventricles were isolated and washed in ice-cold PBS to remove blood cells. Tissues were digested in Hank’s Balanced Salt Solution plus 0.13 U/ml Roche Liberase DH (Thermo Fisher Scientific, Waltham, MA # 5401054001) at 37 °C, while stirring gently with a magnetic spinbar. Supernatants were collected every 5 minutes and neutralized with 10% sheep serum. Dissociated cells were spun down, washed in PBS, and resuspended in PBS plus 2% fetal bovine serum. The cell suspension was then strained through a 35 μm mesh and DAPI added as a viability dye. Sorting was performed on a FACSAria II (BD Biosciences, Franklin Lakes, NJ), gated to exclude doublets, debris, and dead cells. Viable cells were sorted directly into Ambion TRIzol LS Reagent (Invitrogen - Thermo Fisher Scientific, Waltham, MA # 10296–010). RNA was extracted using Zymo Direct-zol RNA Microprep kit, (Zymo Research Corp, Irvine, CA #R2069) as directed by the manufacturer. Total RNA was amplified with the Ovation® PicoSL WTA Kit (NuGEN, Redwood City, CA #3312) to generate cDNA for downstream qPCR analysis.

### Enzyme-Linked Immunosorbent Assay (ELISA) for PGE_2_

ELISA for PGE_2_ was conducted using the Prostaglandin E_2_ Human ELISA Kit (Invitrogen - Thermo Fisher Scientific, Waltham, MA # KHL1701) according to manufacturer’s instructions. Briefly, ventricles were collected from six weight-matched clutchmates, washed in ice-cold PBS, and homogenized in 500 μl TRIS buffer. Homogenate was spun down at 1200 rpm for four min at 4 °C to eliminate particulate, and supernatant collected for ELISA. Assays were run in technical triplicate.

### LC-MS/MS analysis: Lipid isolation

For each biological replicate, ventricles from five weight-matched clutchmates were isolated, washed in ice-cold PBS, flash frozen in liquid nitrogen and stored at −80 °C until shipment on dry ice to the University of Iowa for sample preparation and analysis; samples were processed and analyzed in a blinded manner. Tissues were homogenized in 1 ml sucrose buffer (250 mM sucrose, 50 mM HEPES pH 7.1, 1 mM EDTA, 1 mM EGTA with fresh protease inhibitors) using a Dounce homogenizer. A small aliquot was set aside to quantify proteins for sample normalization. Protein was precipitated and separated from homogenates with the addition of 2 ml HPLC grade methanol (Fisher Scientific, Hampton, NH) and centrifugation at 2,200 × g at 4 °C for 15 minutes. Supernatants containing lipids were diluted with 12.5 ml HPLC grade H_2_O (Fischer Scientific, Hampton, NH), and supplemented with 100 μl of internal standard stock including the following deuterated eicosanoids (Cayman Chemical, Ann Arbor, MI): 0.2 ng/μl d8 arachidonic acid, 0.1 ng/μl d4 6-keto-prostaglandin (PG)F_1α_, 0.1 ng/μl d4 PGD_2_, 0.1 ng/μl d4 PGE_2_, 0.1 ng/μl d4 PGF_2α_, 0.1 ng/μl d4 thromboxane (TX)B_2_, 0.1 ng/μl d4 15-deoxy-Δ12,14-PGJ_2_, 0.1 ng/μl d8 5-(S)-hydroxyicosatetraenoic acid (HETE), 0.1 ng/µl 15(S)-HETE, and 0.1 ng/µl 12(S)-HETE diluted in 50% HPLC grade ethanol (Fisher Scientific, Hampton, NH). Samples were loaded onto a Strata-X 33 u Polymeric Reversed Phase column 60 mg/3 ml (Phenomenex, Torrance, CA) that was previously conditioned with HPLC grade methanol and equilibrated with HPLC grade water. Lipids were washed with HPLC grade water and eluted in HPLC grade methanol. Eluate was dried using a SpeedVac and samples were suspended in 100 μl of liquid chromatography (LC) solvent A (water-acetonitrile-formic acid (63:37:0.02, v/v/v)) for LC-MS/MS analysis. Samples were stored at −80 °C until analyzed.

### Liquid chromatography and mass spectrometry

An ACQUITY UPLC BEH C18 column, 130 Å, 1.7 μm, 2.1 mm × 100 mm with in-line filter (Waters, Milford, MA) was equilibrated at 35 °C at a flow rate of 300 μl/min with LC solvent A on an ACQUITY UPLC H-Class system (Waters, Milford, MA). A small portion of each sample, 30 μl, was loaded onto the column at a flow rate of 300 μl/min of 100% LC solvent A followed by a linear gradient to 20% LC solvent B (acetonitrile-isopropanol(50:50, v/v)) over six min, then increased to 55% LC solvent B over 0.5 min and 100% LC solvent B over 5.5 min and held for four min. Column was primed with 100% LC solvent A for three min between samples. Metabolites were directed to an ACQUITY TQ detector mass spectrometer equipped with an electrospray ionization source set to negative ion mode (Waters, Milford, MA). Specific analytes were detected via multiple reaction monitoring and quantified using a standard curve of known concentration using MassLynx software (Waters, Milford, MA). Each standard (Cayman Chemical, Ann Arbor, MI) and internal standard was resolved at the follow retention time (RT) with the following mass transitions: arachidonic acid, RT 12.52 min, m/z 303 → 259; 6-keto- PGF_1α_, RT 1.47 min, m/z 369 → 207; PGD_2_, RT 3.42 min, m/z 351 → 189; PGE_2_, RT 2.94 min, m/z 351 → 189; PGF_2α_, RT 2.63 min, m/z 353 → 193; TXB_2_, RT 2.12 min, m/z 369 → 169; 15-deoxy-Δ12,14-PGJ_2_, RT 8.62 min, m/z 315 → 271; 5-(S)-HETE, RT 9.58 min, m/z 319 → 115; 12-(S)-HETE, RT 9.24 min, m/z 319 → 179; 15-(S)-HETE, RT 9.15 min, m/z 319 → 175; d8 arachidonic acid, RT 12.45 min, m/z 311 → 267; d4 6-keto- PGF_1α_, RT 1.46 min, m/z 373 → 211; d4 PGD_2_, RT 3.36 min, m/z 355 → 193; d4 PGE_2_, RT 2.95 min, m/z 355 → 193; d4 PGF_2α_, RT 2.51 min, m/z 357 → 197; d4 TXB_2_, RT 2.13 min, m/z 373 → 173; d4 15-deoxy-Δ12,14-PGJ_2_, RT 8.61 min, m/z 319 → 275; d8 5-(S)-HETE, RT 9.55 min, m/z 317 → 116. Results were further normalized to sample protein concentrations using Microsoft Excel (Microsoft, Redmond, WA).

## Results

### Cardiac injury triggers an elevation in PGE_2_

**Figure 1:**
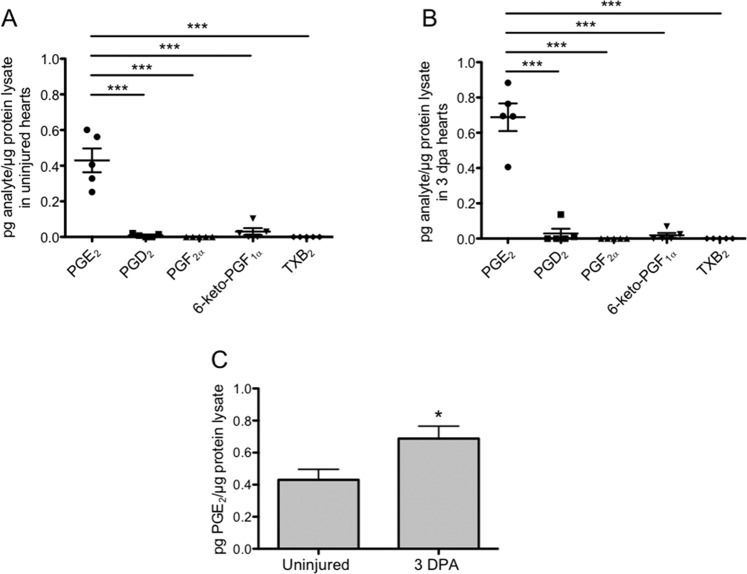
Cardiac injury triggers an elevation in PGE_2_ synthesis. (**A**,**B**) LC-MS/MS profiling showed that PGE_2_ concentrations were significantly elevated above all other prostaglandin species analyzed in both the uninjured heart (**A**), and at 3 dpa (**B**). (mean ± s.e.m. n = 5 biological replicates; 5 pooled ventricles per replicate. One-way ANOVA followed by Tukey’s multiple comparisons test. ***P < 0.001). (**C**) LC-MS/MS analysis showed that PGE_2_ concentrations were significantly higher at 3 dpa, relative to uninjured hearts. (mean ± s.e.m. n = 5 biological replicates; 5 pooled ventricles per replicate. Student’s t-test. *P < 0.05).

Prostaglandins are powerful lipid signals synthesized at the site of injury that regulate the inflammatory response. To profile cardiac prostaglandins and quantify changes associated with injury, we used Liquid Chromatography Tandem Mass Spectrometry (LC-MS/MS). In uninjured hearts, PGE_2_ concentrations were significantly higher than those detected for other prostanoids. PGD_2_ and 6-keto PGF_1α_, a stable metabolite of prostacyclin, barely registered above background levels, while TXB_2_, a Thromboxane A metabolite, and PGF_2α_, were undetected (Fig. 1A). To define prostaglandin levels during regeneration, we amputated ~20% of the adult ventricle, allowed regeneration to proceed for 3 days, and extracted ventricles for analyses. At 3 days post-amputation (dpa), concentrations of PGE_2_ were again, significantly higher than all other prostanoids examined (Fig. 1B). Furthermore, we found that at 3 dpa, concentrations of PGE_2_ increased by more than a 60% relative to uninjured hearts (Fig. 1C). Together, these experiments identify PGE_2_ as the most abundant prostanoid in the zebrafish heart, and establish an injury-induced increase in PGE_2_ concentrations.

### Enzymes critical to PGE_2_ synthesis are upregulated in regenerating adult hearts

Having shown that PGE_2_ is elevated in the heart after injury, we next asked how enzymes critical to PGE_2_ synthesis are modulated during regeneration. COX enzymes catalyze the first rate-limiting step in prostanoid synthesis. Mammals have two COX isozymes, constitutively expressed COX1, and inducible COX2. Three Cox isozymes have been identified in the zebrafish, a single ortholog of mammalian COX1, and two orthologs of mammalian COX2: Cox2a and -2b^43^. Downstream of the Cox enzymes, prostaglandin E synthase (Ptges), the zebrafish ortholog of mammalian PTGES, catalyzes the terminal step of PGE_2_ biosynthesis^44^.

**Figure 2:**
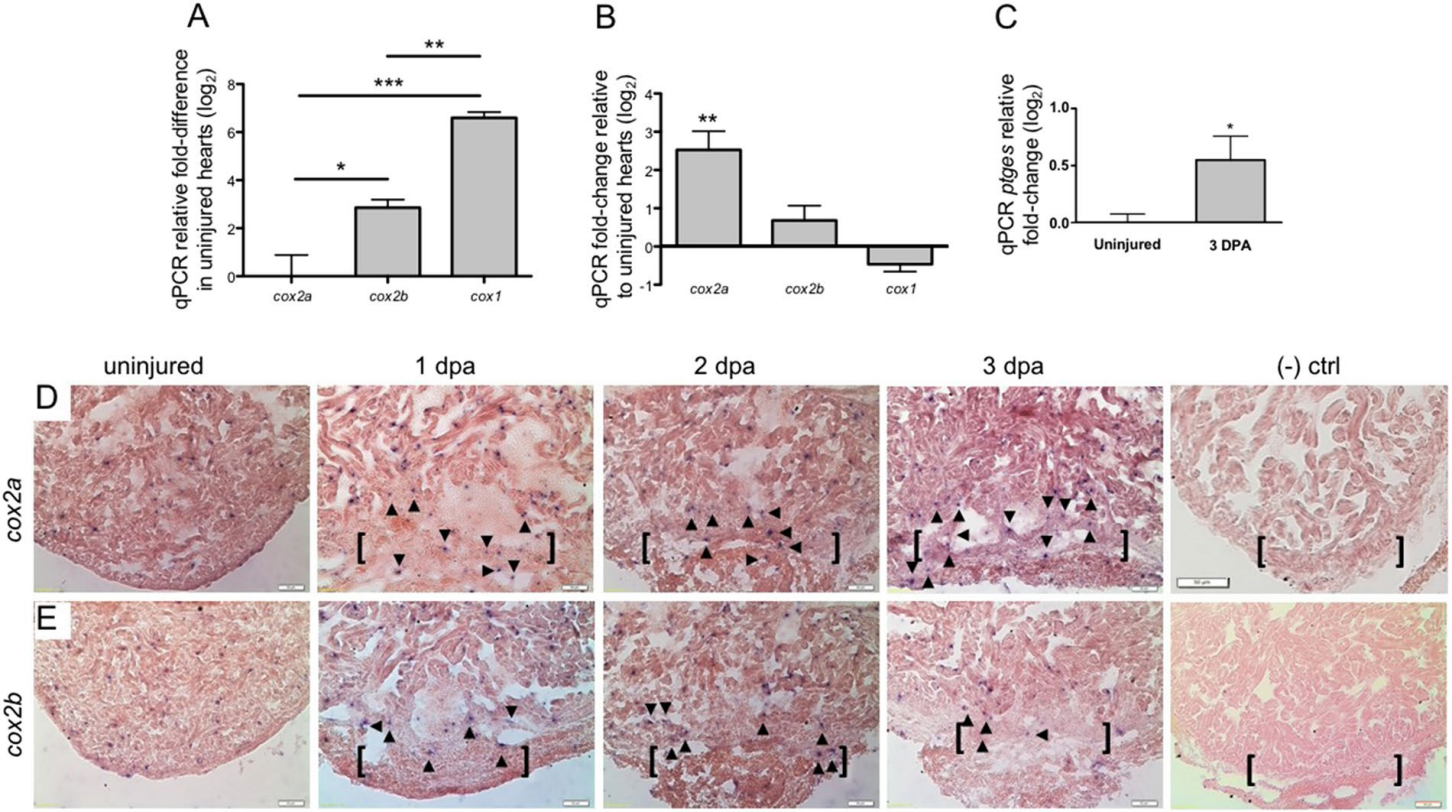
Enzymes critical to PGE_2_ production are upregulated in regenerating adult hearts. (**A**) qPCR studies demonstrated that *cox1* levels in uninjured zebrafish ventricles were significantly higher than either *cox2a* or *cox2b* transcripts. Gene expression was calculated relative to *cox2a* (mean ± s.e.m. n = 4–5 biological replicates; 3–5 pooled ventricles per replicate. One-way ANOVA followed by Tukey’s multiple comparisons test. *P < 0.05, **P < 0.01, ***P < 0.001). (**B**) At 3 dpa, *cox2a* is the only Cox isozyme significantly upregulated relative to uninjured hearts, as determined by qPCR. (mean ± s.e.m. n = 4–5 biological replicates; 3–5 pooled ventricles per replicate. Student’s t-test. **P < 0.01). (**C**) Relative to uninjured hearts, *ptges* is significantly upregulated at 3 dpa. (mean ± s.e.m. n = 4 biological replicates; 3–5 pooled ventricles per replicate. Student’s t-test. *P < 0.05). (**D**,**E**) Representative images of *cox2a* (**D**) and *cox2b* (**E**) expression domains as revealed by *in situ* hybridization studies. (n = 4 biological replicates; brackets = approximate amputation zone; arrowheads demarcate signal within the injury zone). Representative (−) control 2 dpa hearts were hybridized with *cox2a* riboprobe without anti-DIG antibody (top right panel) or with anti-DIG only (bottom right panel). (n = 2 biological replicates).

To characterize relative expression of the Cox enzymes, we conducted qPCR studies in uninjured and 3 dpa regenerating hearts. These assays showed that in uninjured ventricles, *cox1* expression was significantly elevated relative to both *cox2a* (>log_2_ 6.5-fold) and *cox2b* (>log_2_ 2.5-fold) (Fig. 2A). At 3 dpa however, *cox2a* levels had increased more than log_2_ 2.5-fold relative to uninjured hearts. No significant changes were observed in *cox2b* or *cox1* transcripts (Fig. 2B). Mirroring the response of *cox2a*, expression of the terminal prostaglandin synthase *ptges* was also significantly upregulated (>log_2_ 0.5-fold) after amputation injury when compared to uninjured levels (Fig. 2C).

To define the spatial distribution of *cox2a* and *cox2b* during heart regeneration, we performed *in situ* hybridizations on uninjured, 1, 2 and 3 dpa regenerating hearts using DIG labelled RNA probes. While *cox2a* expression was observed in CMs at all time points, expression became enriched within the resection injury zone at 2 and 3 dpa (Fig. 2D). This localized signal suggests potential expression in immune, epicardial and/or endocardial cells. Expression of *cox2b* displayed a more uniform pattern and intensity across the early stages of heart regeneration (Fig. 2E). These signals, however, were absent when heart sections were hybridized with *cox2a* riboprobe in the absence of an anti-DIG antibody (Fig. 2D) or with anti-DIG independently (Fig. 2E).

Together, these results demonstrate that the zebrafish dynamically responds to cardiac injury by upregulating both *cox2a* and *ptges*, two enzymes critical to PGE_2_ synthesis. Importantly, increased expression of these genes correlates directly with increased levels of PGE_2_ observed in ventricles subsequent to injury.

### *Cox2a* expression is highest in epicardial cells at 3 dpa

**Figure 3:**
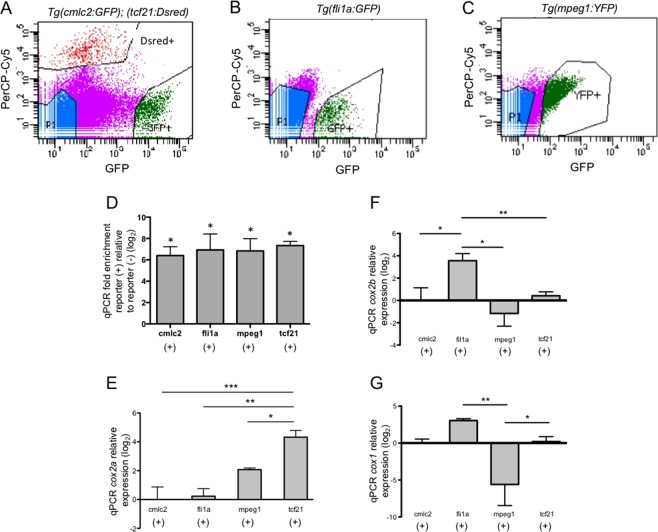
C*ox2a* expression is highest in epicardial cells at 3 dpa. (**A–C**) Representative FACS dot plots displaying gating parameters used to isolate cells from (**A**) *Tg(cmlc2:EGFP); Tg(tcf21:DsRed)*, (**B**) *Tg(fli1a:eGFP)*, (**C**) *Tg(mpeg1:YFP)* reporter lines at 3 dpa. (**D**) qPCR analyses of population specific markers within reporter(+) and reporter(−) cells validate the purity of FACS isolated cells. (mean ± s.e.m. n = 3 biological replicate for each cell type. Student’s t-test *P < 0.05) (**E**) qPCR studies in 3 dpa FACS sorted cells showed *cox2a* expression is significantly higher in tcf21(+) cells, relative to all other cell types examined. (**F**) qPCR of FACS sorted cells showed that at 3 dpa, *cox2b* expression was significantly higher in fli1a(+) cells relative to all other cell types examined. (**G**) There was no significant difference in *cox1* expression among the resident cardiac cells assayed. *cox1* expression in mpeg1(+) cells was significantly lower than that observed in fli1a(+) and tcf21(+) cells. Gene expression was calculated relative to cmlc2(+) cells. (mean = ±s.e.m. n = 2–5 biological replicates; 12–45 pooled ventricles per replicate. One-way ANOVA followed by Tukey’s multiple comparisons test. *P < 0.05, **P < 0.01, ***P < 0.001).

Multiple cell types contribute to the injury-stimulated production of prostaglandins, the end products of Cox activity. To localize expression of the Cox enzymes in the injured heart, and identify potential sites of PGE_2_ synthesis, we utilized Fluorescence-Activated Cell Sorting (FACS) to isolate distinct cell populations for qPCR studies. CMs, epicardial cells, endocardial/endothelial cells, and macrophages were sorted from the dissociated ventricles of *Tg(cmlc2:EGFP); (tcf21:DsRed)*, *Tg(fli1a:EGFP)*, and *Tg(mpeg1.YFP)* strains, respectively (Fig. 3A–C). Purity of the sorts was validated by qPCR, which revealed a log_2_ 6 to 7-fold enrichment of cell-specific markers in fluorescent(+) vs. fluorescent(−) cells (Fig. 3D).

qPCR analysis showed that at 3 dpa, *cox2a* expression was lowest in cmlc2(+) CMs and fli1a(+) endocardial/endothelial cells. Relative to cmlc2(+) cells, we observed a trend towards increased levels of *cox2a* levels in mpeg1(+) macrophages, which have classically been associated with *cox2* expression^45,46^. Unexpectedly, we found *cox2a* expression was ~log_2_ 2-fold higher in tcf21(+) epicardial cells relative to macrophages (Fig. 3E). Expression of c*ox2b*, which showed a muted response to injury, was highest in fli1a(+) cells (Fig. 3F). There was no significant difference in *cox1* expression among the resident cardiac cells assayed; however, *Cox1* expression in mpeg1(+) cells was significantly lower than that observed in fli1a(+) and tcf21(+) cells (Fig. 3G). From these studies, we identified tcf21(+) epicardial cells as the primary site of inducible *cox2a* expression during the early stages of zebrafish heart regeneration.

### Injury activates a differential shift of PGE_2_ receptor expression in the heart

**Figure 4:**
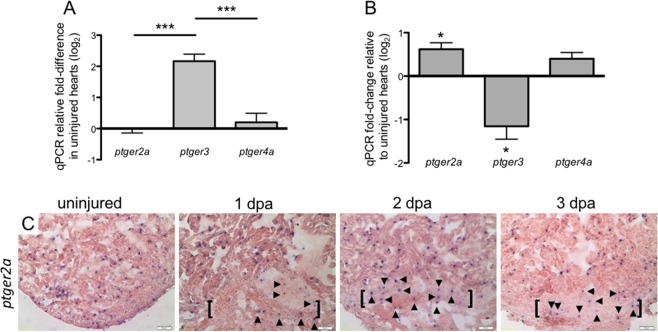
Injury activates a differential shift in PGE_2_ receptor expression in the heart. (**A**) qPCR determination of PGE_2_ receptor expression showed that in uninjured hearts, *ptger3* levels were significantly higher than *ptger2a* or *ptger4a*. Gene expression was calculated relative to *ptger2a*. (mean ± s.e.m. n = 4–5 biological replicates; 3–5 pooled ventricles per replicate. One-way ANOVA followed by Tukey’s multiple comparisons test. ***P < 0.001). (**B**) Relative to uninjured hearts, expression of *ptger2a* was significantly upregulated, while *ptger3* was significantly downregulated at 3 dpa. (mean ± s.e.m. n = 5 biological replicates; 3–5 pooled ventricles per replicate. Student’s t-test. *P < 0.05). (**C**) Representative *in situ* hybridization images of *ptger2a* expression in uninjured, 1, 2 and 3 dpa hearts. (n = 4 biological replicates, brackets mark approximate amputation zone; arrowheads demarcate signal within the injury zone).

Having shown that the zebrafish heart responds to injury by upregulating the expression of *cox2a* and downstream production of PGE_2_, we next examined the expression of PGE_2_ receptors. Mammals express four G protein-coupled PGE_2_ receptors, EP1, EP2, EP3, and EP4. Zebrafish orthologs of the mammalian EP1-4 receptors are Ptger1a, Ptger2a, Ptger3, and Ptger4a, respectively. Examination of receptor expression in the uninjured heart demonstrated that the level of *ptger3* was higher by a log_2_ factor of ~2 relative to both *ptger2a* and *ptger4a* (Fig. 4A). *Ptger1a* was undetected in the ventricles (data not shown).

Injury induced a differential shift in receptor expression, and at 3 dpa, *ptger2a* was upregulated by more than log_2_ 0.6-fold while *ptger3* was downregulated by more than log_2_ 1-fold relative to uninjured hearts. We observed no significant change in expression for *ptger4a* (Fig. 4B). *In situ* hybridization studies revealed *ptger2a* expression was similar to the spatial dynamics of *cox2a* levels. *Ptger2a* was expressed throughout the heart and enriched within the wounded apex in 1, 2 and 3 dpa hearts (Fig. 4C). These results demonstrate that in the zebrafish, cardiac injury activates a dynamic shift in PGE_2_ receptor expression, upregulating the proliferation-associated EP2 ortholog *ptger2a*, while downregulating the functionally opposing receptor, *ptger3*.

### Activation of the Cox2-PGE_2_ circuit stimulates cardiomyocyte proliferation

**Figure 5:**
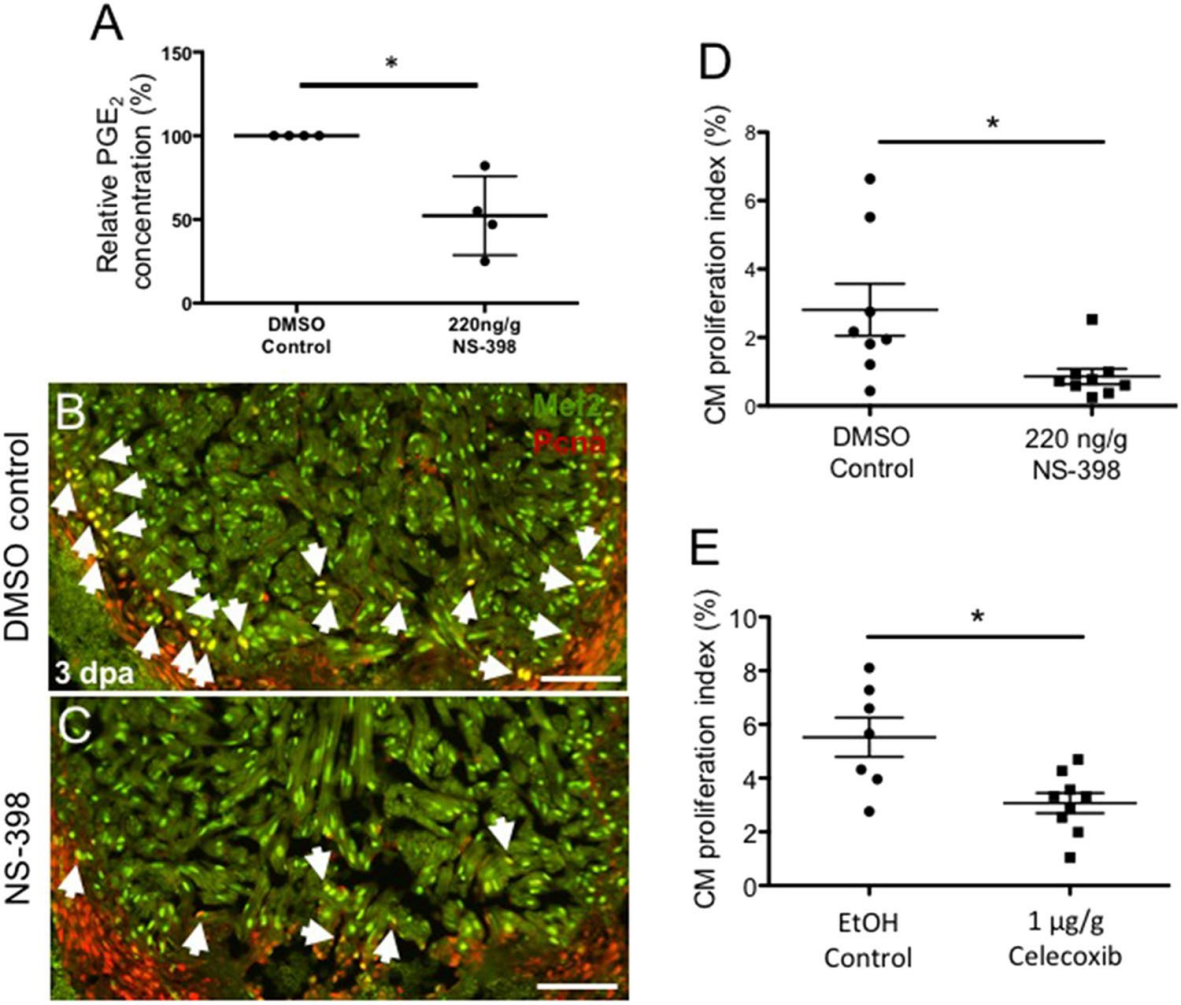
Activation of the Cox2-PGE_2_ circuit stimulates cardiomyocyte proliferation. Zebrafish were subjected to ventricular amputation and treated with daily intraperitoneal (IP) injections of either vehicle control, NS-398 or Celecoxib. Hearts were collected for analysis at 3 dpa. (**A**) Treatment with NS-398 reduced PGE_2_ concentrations in ventricles by ~47%, relative to DMSO controls. (mean ± s.e.m. n = 4 biological replicate for each group; 6 pooled ventricles from weight-matched clutch mates per replicate. Student’s t-test. *P < 0.05). (**B**,**C**) Representative images of 3 dpa injured hearts treated daily with a DMSO control (**B**) or NS-398 (**C**). Hearts were stained with Mef2 (green), and Pcna (red). Mef2+Pcna+ cells mark proliferating CMs, highlighted by white arrows. Scale bars represent 50 μM. (D-E) CM proliferation indices were calculated as a percentage of Mef2(+)Pcna(+) cells relative to the total number of Mef2(+) cells in a defined area adjacent to the injury. (**D**) CM proliferation was reduced by more than 69% in animals treated with NS-398 when compared to controls. (**E**) Reduction of CM proliferation was greater than 54% in animals treated daily with Celecoxib, relative to controls. (mean ± s.e.m. n = 7–9 hearts from clutchmates; three sections were quantified per heart and results averaged. Student’s t-test. *P < 0.05).

PGE_2_ has been shown to promote cell proliferation in multiple contexts. To better understand the impact of Cox2 activity on PGE_2_ synthesis and CM proliferation, we subjected animals to ventricular amputation and treated them with either NS-398, a small molecule shown to selectively inhibit Cox2 activity in the zebrafish^47^, or DMSO control. At 3 dpa, ELISA assays demonstrated that PGE_2_ concentrations were reduced by more than 47% in NS-398 treated hearts relative to controls (Fig. 5A). To determine effects of suppressed Cox2 activity upon heart regeneration, we quantified CM proliferation indices at 3 dpa, a time during regeneration previously demonstrated to exhibit high levels of proliferative activity^48^. Immunostaining for Myocyte enhancer factor −2 (Mef2-green), a nuclear CM marker, and Proliferating cell nuclear antigen (Pcna-red), a nuclear marker for proliferating cells, showed that NS-398 treatment suppressed CM proliferation by approximately 70% relative to controls at 3 dpa (Fig. 5B–D). To confirm these findings, we examined the effects of another selective Cox2 inhibitor, Celecoxib. At 3 dpa, Celecoxib treatment also had a significant effect, reducing CM proliferation indices by almost 50% relative to controls (Fig. 5E). Collectively, these studies demonstrate that, in the injured zebrafish heart, Cox2 activity drives both PGE_2_ synthesis and CM proliferation during the early stages of heart regeneration.

## Discussion

Prostaglandin E_2_ (PGE_2_) is a potent inflammatory mediator, with pleiotropic effects. In the present study, we have identified a pro-regenerative role for PGE_2_ during cardiac regeneration in adult zebrafish. After ventricular amputation, the injured zebrafish heart revealed increased levels of PGE_2_, as well as upregulated expression of *cox2a* and *ptges*, two enzymes critical to PGE_2_ synthesis (Figs. 1, 2). Importantly, pharmacologic inhibition of Cox2 activity suppressed both PGE_2_ and CM proliferation, supporting a crucial role for the Cox2-PGE_2_ circuit in initiating the regenerative response (Fig. 5).

In addition to the wound environment, we unexpectedly identified the epicardium as a potential source of inflammation-associated prostaglandin signaling in the regenerating heart. During the inflammatory response, inducible Cox2 has canonically been associated with immune cells recruited to the site of injury, notably macrophages^19^. However, during conditions of homeostasis, Cox2 is constitutively maintained in some tissues including the vascular endothelium, where it supports the synthesis of prostanoid vasodilators^49,50^. The zebrafish genome encodes two Cox2 genes, Cox2a and −2b. During regeneration, FACS studies revealed expression of the most inducible Cox enzyme, *cox2a*, was highest in the epicardium, while more constitutively expressed *cox2b* was most prominent in endocardial/endothelial cells (Fig. 3). These findings raise the intriguing possibility that Cox2a and Cox2b have divergently evolved in specific tissues, such that Cox2b is expressed in endothelial cells to maintain vascular tone, while in the epicardium, Cox2a facilitates a rapid inflammatory response.

An emerging role for the epicardium as a source of inflammatory signaling has been highlighted in both mammalian and zebrafish models. In mice, epicardial cells were found to be a source of YAP-mediated IFNγ production following myocardial infarction, orchestrating the recruitment of immune-suppressive regulatory T cells (T_regs_)^11^. PGE_2_ has been implicated in both YAP activation^36^, and T_reg_ recruitment^17^, suggesting that in the epicardium, crosstalk between these pathways could mediate the inflammatory response. Our current work supports this study, identifying the epicardium as a potential locus of reparative, inflammatory signaling.

In comparison to our qPCR studies (Figs. 2B and 4B), *in situ* hybridizations revealed more uniform expression for *cox2a*, *−2b* and *ptger2a* in response to injury (Figs. 2D,E and 4C). One potential explanation for the expression differences observed between these two methodologies is their relative sensitivity. Highly sensitive qPCR assays are more likely to amplify subtle differences in transcript levels, compared to the semi-quantitative nature of *in situ* hybridizations. Interestingly, we also noted that while *in situ* studies identified *cox2* transcripts in both injury and remote zones of the adult heart, CM proliferation appeared to be limited to the wound area (Fig. 5B). It is possible this localized proliferation may be attributable to additional factors, either related, or unrelated to Cox2 activity, that are spatially restricted during regeneration.

In the downstream PGE_2_ signaling circuit, we found that PGE_2_ receptor expression was dynamically modulated in the regenerating heart. PGE_2_ signals through the autocrine and paracrine activation of four G protein-coupled receptors to initiate diverse downstream pathways. Activation of EP2, the ortholog of zebrafish Ptger2a, directly promotes cell proliferation in the context of regeneration and cancer^23,51^, polarizing neutrophils and macrophages towards a reparative phenotype^18,19^. By contrast, EP3, the ortholog of zebrafish Ptger3, appears to activate pathways in opposition to EP2, inhibiting cell proliferation^25,26^ and exacerbating pathologic inflammation^13,52^. Our profiling studies of PGE_2_ receptor expression revealed that injury drives the upregulation of *ptger2a* and reciprocal downregulation of *ptger3* in cardiac tissues (Fig. 4), suggesting that in the zebrafish heart, PGE_2_ signaling is directed towards a restorative response.

Whether modulation of the PGE_2_ circuit after injury has an enduring impact on cardiac regeneration remains an open question. Our work showed that in the regenerative zebrafish heart, injury triggers the upregulation of genes critical to PGE_2_ synthesis (Fig. 2). We furthermore demonstrated that activation of the PGE_2_ signal is essential to stimulate CM proliferation, a major driver of heart regeneration (Fig. 5).

In other model systems, PGE_2_ has been shown to directly promote the proliferation of multiple cell types, including skeletal muscle stem cells^28^ and primary human CMs^15^. However, a large body of work also supports a role for PGE_2_ in governing the recruitment, retention, and pro-regenerative polarization of neutrophils, macrophages, and T cells^17,18,53^. Therefore, it is likely that PGE_2_ stimulates CM proliferation directly, as well as indirectly, through orchestrating restorative immune cell activity. This study, documenting the injury-induced modulation of PGE_2_signaling during cardiac regeneration, seeds the field for future research to define the mechanisms through which PGE_2_ promotes the early, regenerative response.
